# A Continuously Worn Dual Temperature Sensor System for Accurate Monitoring of Core Body Temperature from the Ear Canal

**DOI:** 10.3390/s23177323

**Published:** 2023-08-22

**Authors:** Kyle D. Olson, Parker O’Brien, Andy S. Lin, David A. Fabry, Steve Hanke, Mark J. Schroeder

**Affiliations:** Starkey, 6600 Washington Ave S., Eden Prairie, MN 55344, USA; kyle_olson@starkey.com (K.D.O.); parker_obrien@starkey.com (P.O.); andy_lin@starkey.com (A.S.L.); dave_fabry@starkey.com (D.A.F.); steve_hanke@starkey.com (S.H.)

**Keywords:** core body temperature, ear, hearable, hearing aid, temperature measurement

## Abstract

The objective of this work was to develop a temperature sensor system that accurately measures core body temperature from an ear-worn device. Two digital temperature sensors were embedded in a hearing aid shell along the thermal gradient of the ear canal to form a linear heat balance relationship. This relationship was used to determine best fit parameters for estimating body temperature. The predicted body temperatures resulted in intersubject limits of agreement (LOA) of ±0.49 °C over a range of physiologic and ambient temperatures without calibration. The newly developed hearing aid-based temperature sensor system can estimate core body temperature at an accuracy level equal to or better than many devices currently on the market. An accurate, continuously worn, temperature monitoring and tracking device may help provide early detection of illnesses, which could prove especially beneficial during pandemics and in the elderly demographic of hearing aid wearers.

## 1. Introduction

The measurement of body temperature is as important and prevalent today as at any time in our past. Body temperature provides important insight on the current health of the body, such as whether the body is trying to fight off an infection or is overheating due to excessive exercise. In the last couple of years, the monitoring of body temperature has taken center stage as a useful tool in many businesses and medical facilities for quickly screening people for the COVID-19 virus [1,2]

For people with hearing loss, however, measuring body temperature may be especially useful because, while hearing loss itself is not typically associated with elevated body temperature, there are numerous comorbid health conditions that may be associated with both hearing loss and elevated body temperature. These include:

- Infections: Various infections, such as ear infections, sinusitis, tonsillitis, and pneumonia, can cause both hearing loss and fever. Infections are a common cause of fever and can lead to temporary or permanent hearing loss if left untreated [3].
- Autoimmune disorders: Certain autoimmune disorders, such as rheumatoid arthritis, lupus, and multiple sclerosis, can cause inflammation throughout the body, which can lead to both hearing loss and fever [4].
- Meniere’s disease: Meniere’s disease is a disorder of the inner ear that can cause vertigo, hearing loss, and tinnitus. In some cases, it can also cause fever [5].
- Ototoxicity: Certain medications, such as some antibiotics and chemotherapy drugs, can cause both hearing loss and fever as a side effect [6].
- Heatstroke: Exposure to high temperatures for an extended period can lead to heatstroke, which is a medical emergency. Symptoms of heatstroke include fever, rapid heartbeat, and confusion, and it can cause hearing loss or other complications if left untreated [7].

Unfortunately, one-time temperature measurements are not ideal, whether for individual purposes, COVID-19 screening, or remote patient monitoring. Since fevers can go through phases, a single temperature measurement might miss the presence of a fever. Additionally, numerous factors can affect a person’s temperature, including medications, the environment, exercise, skin color, gender, race, and body temperature variations throughout the day and month [8,9,10,11]. Moreover, there are differences in people’s individual basal body temperatures, which presents an even greater challenge in older adults due to increased variation in their baseline temperatures [12].

Thus, single temperature measurements do not provide the full picture of an individual’s temperature profile and can, therefore, easily misclassify a person as febrile or afebrile when based solely on an over-generalized reference point such as 38 °C. This provides an argument that continuous tracking of temperature over time is more useful than using an isolated measurement. This is true whether conducting remote monitoring or evaluating a person for an illness such as influenza or COVID-19. Additionally, continuous temperature measurements can help provide the earliest warning for the presence of fever.

Accuracy and convenience have long been two key objectives when it comes to measuring core body temperature. Unfortunately, meeting one objective generally comes at the expense of the other. For example, the pulmonary artery may provide the most accurate core body temperature, but it is certainly not as convenient as measuring skin temperature, one of the least accurate measurement sites. Fortunately, numerous options with varying accuracy and convenience exist for people to measure their body temperature. These include liquid-in-glass and digital thermometers, for oral, rectal, and armpit measurements, and infrared thermometry for use on the forehead and inside the ear canal.

However, although oral and infrared in-ear thermometers are widely used devices, continuously worn in-ear temperature measurement systems that provide convenient temperature tracking have yet to be heavily adopted. This may be due in large part to the paucity of such devices in the consumer market. The reason for their scarcity may be due to a few key factors such as (1) lack of demand due to consumers not fully recognizing the benefits of continuously monitoring core body temperature (CBT), (2) manufacturers not identifying an acceptable platform upon which to merge such temperature measuring technology, and (3) difficulties in developing a CBT measurement device that can meet the accuracy, size, comfort, and convenience expected by consumers.

Recently, the ear has been recognized as a prime location to exploit for the purpose of health biometrics [13,14,15,16,17]. Building on that sentiment, the first two challenges outlined above can be addressed by integrating temperature measurement into devices that are already commonly worn by consumers, such as hearing aids and earbuds. Augmenting an existing product would provide convenient health monitoring of an important vital signal that can be performed unobtrusively without user intervention. When paired with a smartphone that can record and track the temperature readings over extended periods, the temperature measurement system could provide important physiological information that has rarely been available to general consumers, or even medical personnel. 

The last challenge regarding accuracy, size, comfort, and convenience must be addressed through careful design, development, and validation of a temperature measurement system. Of utmost importance is the ability to estimate physiological temperatures with an accuracy on a par with “gold standard” methods used to measure core temperature. Typically, that accuracy is defined as having limits of agreement (LOA) of ±0.5 °C when compared to approved CBT measuring methods and locations [18]. The locations most accepted for accurately measuring CBT are the pulmonary artery, rectum, esophagus, and bladder. However, owing to the invasiveness and inconvenience associated with these measurement locations, other acceptable reference temperature locations and methods include sublingually using an electronic or liquid-in-glass thermometer, the forehead using zero-heat-flux (ZHF) thermometry, and the tympanic membrane using infrared thermometry.

In an attempt to provide a continuous and convenient temperature measurement device, while also maintaining a high level of accuracy, an in-ear, dual temperature sensor measurement system has been developed. The sensors were integrated into the receiver of a hearing aid developed by Starkey, a prominent hearing aid manufacturer. This paper describes the theory, design and experimental evaluation of the hearing aid-based temperature measurement system.

## 2. Numerical and Experimental Methods

### 2.1. Heat Balance Model Theory

The tympanic membrane is often used to estimate core body temperature, primarily through the use of infrared thermometry [19]. Since it is impractical to place a continuously worn sensor of any kind at the tympanic membrane, the sensor must be placed closer to the canal entrance. However, a temperature gradient exists along the ear canal from the tympanic membrane to the outer ear, primarily due to ambient air temperature and wind [19,20]. To help account for this gradient and to make temperature estimates less sensitive to sensor placement, a second sensor can be placed along the gradient. A model of the dual sensor configuration can then be developed to minimize ambient effects and improve the overall accuracy of core body temperature estimates in various ambient settings. 

**Figure 1:**
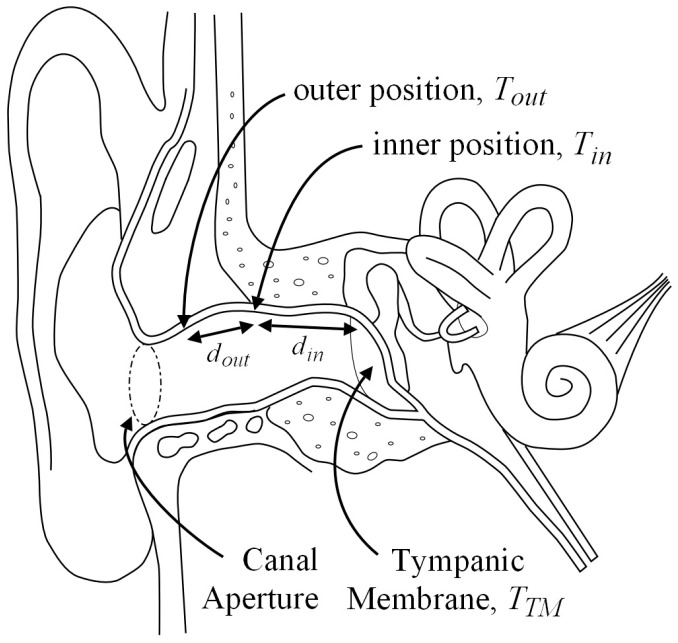
Location of temperature readings for the heat balance model.

Assuming a one-dimensional model of heat flow through the ear canal as shown in Figure 1, the heat flux between the tympanic membrane and the inner canal measurement location can be given by:

(1) $$q_{in}=\frac{K_{in}}{d_{in}}(T_{TM}-T_{in}),$$

where *T_TM_* is the temperature nearest the tympanic membrane, *T_in_* is the inner location temperature, *K_in_* is a composite thermal conductivity of skin, ear wax, plastic, and air between the tympanic membrane and inner location, and *d_in_* is the distance between the tympanic membrane and inner location. Similarly, the heat flux between the inner and outer locations is given by:

(2) $$q_{out}=\frac{K_{out}}{d_{out}}(T_{in}-T_{out}),$$

where *T_out_* is the outer temperature, *K_out_* is another composite thermal conductivity between the inner and outer temperature sensor locations, and *d_out_* is the distance between the inner and outer locations. 

At steady state, the two heat fluxes are equal, resulting in:

(3) $$\left(T_{in}-T_{out}\right)=\frac{K_{in}d_{out}}{K_{out}d_{in}}\left(T_{TM}-T_{in}\right).$$

Therefore, a plot of (*T_TM_ − T_in_*) versus (*T_in_ − T_out_*), or any core temperature reference such as oral temperature instead of *T_TM_*, should result in a linear relationship when the ear and the environment are in thermal steady state. Notice that the terms that are difficult to measure (intersubject ear canal dimensions and thermal conductivity) can now be found empirically by equating the slope of the resulting linear fit. In addition, it should be noted that the linear fit to the data may have a non-zero offset, unlike Equation (3). A non-zero offset arises in the real world due to external heat sources or sinks within the system, such as microprocessors, copper wire, blood flow, and other physiological effects. Including *slope* and *offset* into the model results in the equation:

(4) $$\left(T_{in}-T_{out}\right)=slope\left(T_{TM}-T_{in}\right)+offset,$$

where *slope* is equivalent to the term $K_{in}d_{out}/K_{out}d_{in}$ and *offset* is added for the constant heat flux unaccounted for in the simple model of Equation (3).

Using a linear fit of the data, the estimated core body temperature can then be calculated using the following equation, where only the measured temperatures, *T_in_* and *T_out_*, are required.

(5) $$T_{TM}=T_{estimate}=\frac{\left(T_{in}-T_{out}-offset\right)}{slope}+T_{in}$$

The linear model, being derived from a 1-dimensional system, is expected to be different than a multidimensional model. In fact, the temperature gradient in the ear is not linear. However, Equation (5) is predicting that the ratio of temperature differences from *T_TM_* to *T_in_* and from *T_in_* to *T_out_* will remain constant over time. This relationship will also hold true in a 3D model where the thermal conductivity and distances remain constant. However, the ratio would be more complicated than that shown in Equation (3). Nonetheless, the ratio would be reduced to the *slope* term that can be empirically found through measurement.

A few factors could introduce inconsistencies when using Equation (5). First, if *K_in_* and/or *K_out_* are not constant along the distance between the sensor locations, a nonlinearity in the temperature vs. distance plot is expected. However, if the assumption holds true that these thermal conductivities are time-invariant, then their ratio in Equation (3) will remain constant regardless of any changes over distance. Second, on a population of humans, *K_in_* and *d_in_* may vary, thus their linear relationship could also be different. However, we can partially control *K_out_* and *d_out_* through design, assembly, and manufacturing techniques to reduce the variability across the population.

### 2.2. Equipment Set-Up

#### 2.2.1. Digital Temperature Sensor Selection and Assembly

The digital temperature sensor used in the hearing aid and as a reference in the mouth was the 6-pin TMP117M chip from Texas Instruments (Dallas, TX, USA). The chip has a size of 1.53 mm × 1.00 mm, a low quiescent current of 3.5 µA, and can operate at a low voltage of 1.7 V. Temperature transduction is performed via an internal PNP transistor. Additionally, all signal conditioning and 16-bit analog-to-digital conversion are performed on-chip. The chip outputs a pre-calibrated temperature reading via a digital signal using the I^2^C communication protocol with a worst-case accuracy of ±0.1 °C over a temperature range of −20 °C to 50 °C, or typically less than ±0.05 °C in our range of interest. The combination of these specifications and features makes the chip particularly suitable for integration with our hearing aid platform. 

**Figure 2:**
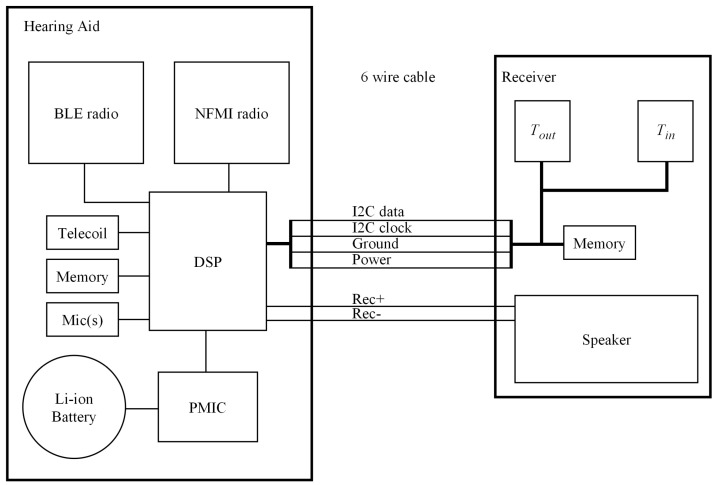
Block diagram of the temperature sensors in the hearing aid.

The temperature sensors were integrated into the custom-made, in-ear, receiver (speaker) portion of a receiver-in-canal style hearing aid. A high-level block diagram of the hearing aid showing the relative placement of the temperature sensors and other major components in the in-ear receiver and behind-the-ear portions is shown in Figure 2. 

After integrating the chips into the hearing aid receiver, a small lot was tested over a 30–45° C temperature range in a calibrated water bath to ensure that our manufacturing and assembly techniques did not affect the manufacturer’s calibration. We found that processing techniques that created stress on the IC package were problematic to keeping the temperature sensors within manufacturing specifications. Some common processing techniques employed to avoid stressing the IC packages were using rigid circuit boards and low temperature solder and avoiding package underfill.

#### 2.2.2. Hearing Device Shell

**Figure 3:**
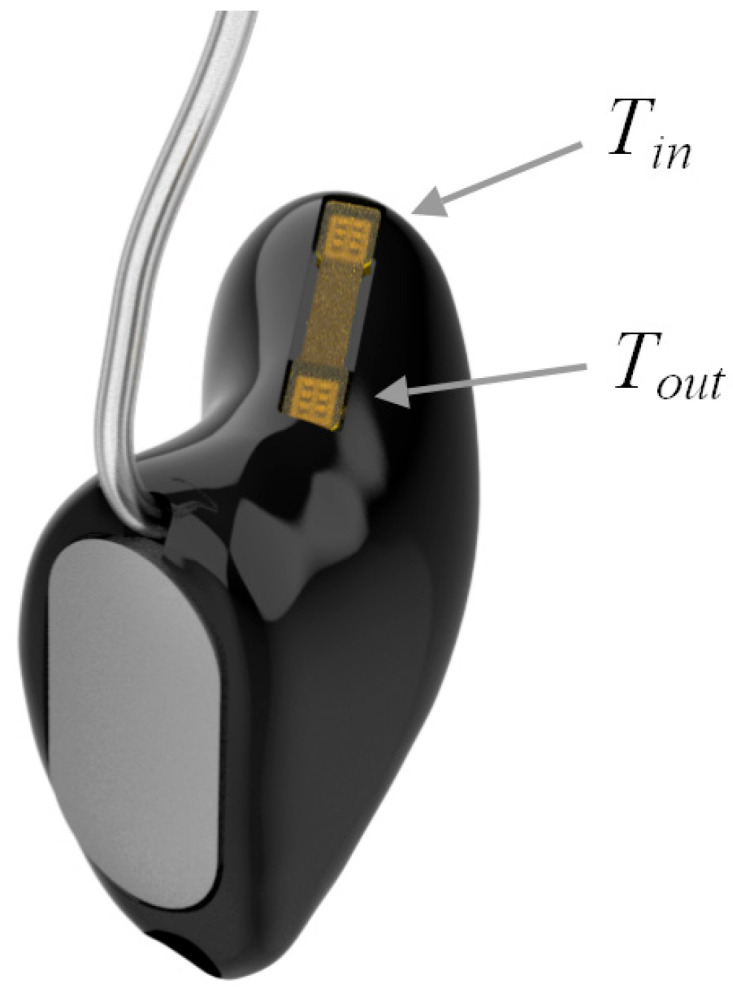
Illustration of a CIC hearing aid shell with two embedded temperature sensors.

Photopolymer custom fit completely-in-canal (CIC) shells were manufactured using 3D additive printing by first laser 3D scanning a negative mold impression of the ear and the ear canal, then using the standard Starkey procedures for 3D modeling and manufacturing of the device. Shells were formed with a sound bore hole at the tip and a vent size commonly used in hearing products to mitigate the occlusion effect and increase comfort of the devices for all-day wear. A cut-out was made on the top side of the shell for mounting the temperature sensors’ flexible printed circuit board (flex PCB). After assembly, the cut-out area was filled with the shell material, totally encasing the flex PCB inside the shell wall. An illustration of the final device assembly is shown in Figure 3.

#### 2.2.3. Data Acquisition and Reference Temperatures

Temperature measurements were acquired from the augmented hearing aids by streaming the data to a smartphone from where it could later be obtained for analysis. Temperature readings were acquired every one second or eight seconds depending on the experiment. To obtain a core body temperature reference, the Starkey hearing aid assembly also had a wired connection to a third digital temperature sensor that was placed in the patient’s mouth under the tongue as far back as was comfortable. The oral sensor was encased in silicone and placed in a disposable sanitary sleeve commonly used for oral thermometers. 

The normal temperature experiments were all performed onsite, whereas the febrile experiments were carried out in an offsite clinical setting. In both cases, the same setup was used to read the in-ear temperature measurements. However, for the febrile experiments, the reference temperature was obtained from either an FDA-cleared and calibrated oral probe (Welch Allyn SURETEMP 692, Skaneateles Falls, NY, USA) or a ZHF forehead patch (Bair Hugger, 3M, St. Paul, MN, USA).

### 2.3. Experiments

All data were collected from human subjects. No health screenings or exclusions of normal living were considered for any experimental subjects. All on-site experiments were carried out following the principles of the Declaration of Helsinki. Off-site experiments were carried out according to protocol PR2020-409, approved by Salus IRB (Austin, TX, USA), 23 December 2020.

Experiments were run at room temperature in an office environment unless otherwise stated. In most cases, in-ear temperature readings were acquired from both the left and right ears of each subject.

#### 2.3.1. Normal Core Body Temperature in Various Ambient Temperature Settings

To test the ability to predict normal CBT, measurements were taken over an ambient temperature range of 5 °C to 32 °C on 31 subjects (9 females, 22 males; age range: 23–65). Room temperature readings were taken in an office environment with the subjects seated. The colder ambient temperature readings were taken with the subjects seated outside on a calm, cool day. The temperature was not controlled, nor were humidity and pressure. However, direct sunlight was not allowed to shine directly on the ears or hearing aids and the receiver shells were sealed and fully encased. 

The variation in thermal contact of the hearing aid shell with the skin due to insertion into the ear was a concern even for the custom devices. Therefore, in order to test the repeatability of results, all subjects in the nonclinical experiment settings completed tests multiple times after removing and reseating the measurement device. 

#### 2.3.2. Febrile Core Body Temperature

A clinical experiment was performed on 11 different subjects (6 female, 5 male; age range: 19–34 years) to test the ability to estimate the body temperature of simulated febrile subjects. Oral and forehead reference temperatures were recorded simultaneously along with left and right in-ear sensor temperatures at room temperature. Each subject was at rest for 20 min prior to recording the baseline temperature. Then, to stop the body from regulating its temperature through sweat, subjects were wrapped in clear plastic cling wrap and placed in a personal sauna. Once the clinical researcher measured a core body temperature around 38 °C, the personal sauna was turned off and the patient was allowed to cool down and remove the plastic cling wrap. Temperature was recorded throughout the entire procedure at 1 Hz but only analyzed at the peak simulated fever for each subject. Therefore, each subject resulted in a maximum of four measurements, a left/right pair of both normal and febrile temperature measurements.

In addition to the eleven subjects with simulated fevers, data were collected from two onsite subjects who possessed natural fevers. In both cases, the digital temperature sensors from the Starkey hearing aid assembly were used to measure the reference temperature.

The data from both experiments were combined and used to generate a Bland–Altman plot and determine the LOA, i.e., the accuracy of the system under test relative to the measured reference temperatures. The LOA is defined as 1.96 times the standard deviation, *s*, of differences between the reference and estimated core body temperatures, $T_{Error}$:

(6) $$s=\sqrt{\frac{\sum_{i=1}^{N}{\left({T_{Error}}_{i}-{\bar{T}}_{Error}\right)}^{2}}{N-1}}$$

where *N* is the number of subjects. An LOA of ±0.5 °C or less was determined to be acceptable since adverse clinical effects are not observed for errors within this range [18].

## 3. Results

### 3.1. Heat Balance Model

**Figure 4:**
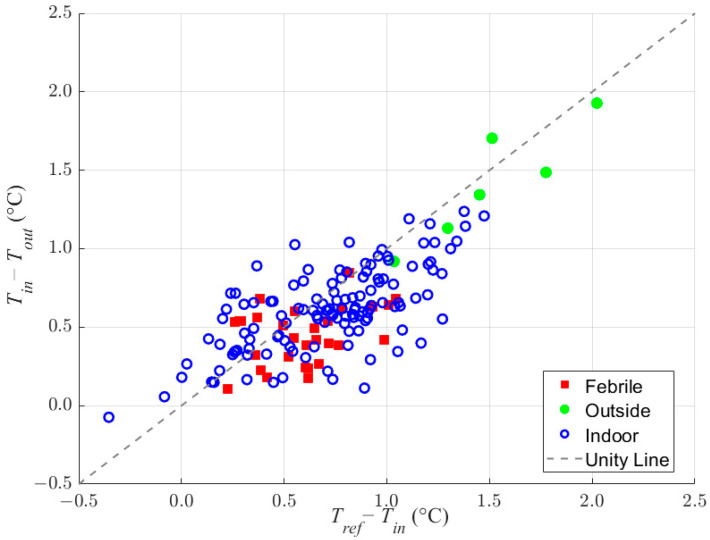
Dual temperature sensor heat balance relationship and identity line indicating near unity linearity.

Figure 4 shows the relationship for a dual temperature sensor heat balance model for 162 unique measurements acquired from sedentary subjects over an ambient range of 5 °C to 32 °C and simulated febrile temperatures of approximately 38 °C. The identity line shown in the figure illustrates that a linear relationship exists between the two heat balance temperature differences regardless of the setting. 

### 3.2. Core Body Temperature Estimation

The data shown in Figure 4 were used to perform a linear fit in the form of Equation (4) to define a temperature prediction model. The slope and offset from the linear fit were then used to estimate the reference temperature, i.e., the core body temperature, for each *T_in_* and *T_out_* measurement pair using Equation (5). 

**Figure 5:**
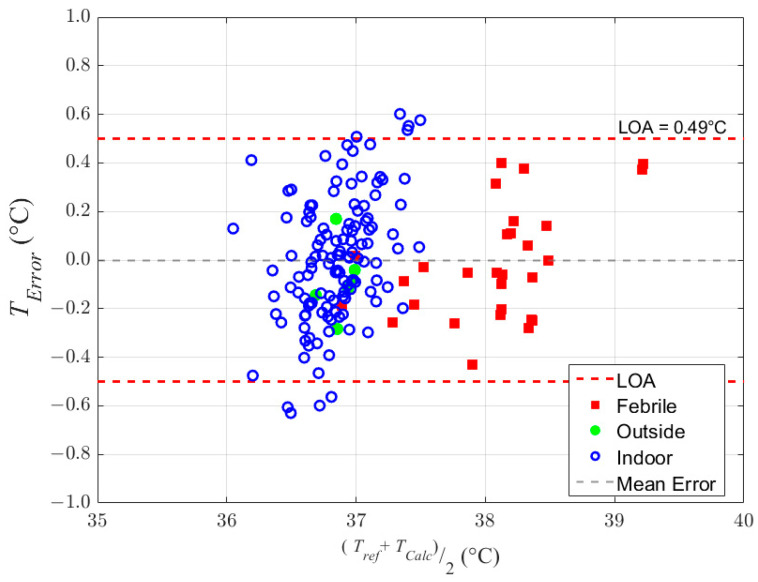
Bland–Altman graph of core body temperature estimates based on the dual sensor heat balance model. Mean error equals zero and the limits of agreement are ±0.49 °C.

The calculated estimates of the reference temperature measurements were evaluated using Bland–Altman analysis [21]. The estimated error of each recording is equal to the difference between the reference temperature (approximately equal to CBT) and the calculated temperature as shown in the Bland–Altman graph of Figure 5 for the 162 measurements. These errors were then used to find the mean error or bias and the LOA for the newly developed core body temperature measurement system. The bias and the LOA were found to be zero and ±0.49 °C, respectively, and all error values fell within ±0.62 °C.

These results compare very favorably with those obtained using only the inner temperature sensor to estimate the reference core body temperature. The single sensor method resulted in a mean bias of 0.73 °C and limits of agreement of ±0.72 °C (results not shown).

Table 1 shows a breakdown of the limits of agreement for the indoor, outdoor, and febrile cases, as well as the combined result.

**Figure 6:**
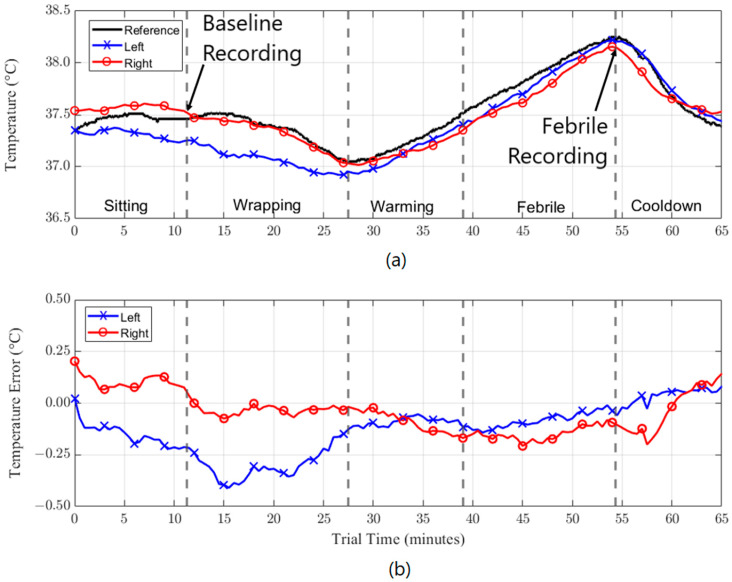
(**a**) Example of reference and estimated core body temperatures (left and right ears) over time for one subject during simulated febrile experiment. (**b**) Error of the estimated temperature over time for left and right sensors.

Figure 6 shows an example of reference and estimated core body temperatures measured over a period of approximately one hour during the simulated febrile study. The core body reference temperature range was 37.05 °C to 38.25 °C. This subject demonstrated an estimated temperature accuracy within ±0.5 °C regardless of the external conditions or activity for the duration of the study for both the left and right sensors. The data points used in the Bland–Altman analysis and shown in Figure 5 consist of measurements recorded at the conclusion of both the sitting and febrile periods. 

## 4. Discussion

The heat balance model resulted in a linear relationship between the reference, inner canal, and outer canal temperatures (Figure 4). This relationship was realized even when vast differences existed between the ambient and reference temperatures, as seen with data collected at 5 °C. The linearity shown throughout the range leads us to believe that the relationship would be maintained at ambient temperatures beyond the tested range. However, additional experiments would need to be performed to verify this expectation. 

As shown in Figure 5 and Table 1, the overall temperature accuracy for all subjects and scenarios combined is ±0.49 °C. When viewed separately, the febrile and outdoor subjects alone have even better LOA accuracies of ±0.45 °C and ±0.29 °C, respectively. This may be due in part to the lower number of subjects and data points in both cases.

As seen in Figure 5, the largest measured error was approximately −0.65 °C. However, a worst-case scenario error of ±1.5 °C can be estimated by using six times the standard deviation, providing the largest expected error in one device per million. Though undesirable, a +1.5 °C error, for example, would not necessarily result in the misreporting of fever. That is because the device would likely always show a similarly large offset error and, therefore, the measured ‘normal’ would likewise be offset as well. Thus, if fever were defined as 1 °C above ‘normal’, the large absolute error would not result in fever being incorrectly reported. This behavior requires that the individual error over numerous reinsertions be much lower than the error across the general population, which we found to be true in our experiments.

The LOA for the described device of ±0.49 °C over a variety of physiological and ambient temperatures indicates an accuracy and robustness that meets the commonly used limit criterion of being less than ±0.5 °C. Additionally, the performance of the developed device exceeds numerous other products on the market. For example, the ear-worn temperature measurement device Cosinuss One (Cosinuss Company, Munich, Germany) demonstrated a bias and LOA of −0.22 ± 0.56 °C and −0.15 ± 0.64 °C when compared to temperatures obtained from a ZHF forehead sensor and bladder catheter, respectively [22]. The Braun Thermoscan IR tympanal ear thermometer (Kaz Europe SA, Lausanne, Switzerland) and the TemporalScanner Model TAT-5000 IR temporal artery thermometer (Exergen Corp., Watertown, MA, USA) showed biases and LOAs of 0.54 ± 0.67 °C and 0.03 ± 0.98 °C, respectively, when compared to rectal CBT measurements [23].

Other dual sensor-type systems in the past have used ZHF theory with stacked thermistor configurations containing a material of known heat transfer coefficient between them to predict core body temperature [24,25]. These resulted in somewhat larger LOAs (±0.53 to ±0.98 °C) than the dual sensor, in-ear system described here. However, the performance of the ZHF forehead thermometer, used as a reference during the clinical febrile experiment in this study, fared better, with bias values near zero and LOAs of ±0.38 °C and ±0.48 °C relative to esophageal measurements in two separate studies with physiologic temperature ranges similar to this study’s temperature range [26,27]. 

The developed temperature sensor’s relatively good performance is likely attributable to a combination of factors. These include the temperature sensors’ proximity to the tympanic membrane, which is an accepted proxy for CBT, and the use of dual temperature sensors possessing a relatively large distance between them that more optimally account for ambient temperature effects. Additionally, the interface of the temperature sensors with the body, as well as the materials used between the sensors, effectively measure the heat flux through the body rather than the heat flux through an external sensor. By measuring the skin temperature at different depths within the ear canal, the sensors are effectively measuring the heat flux from a shallow depth to a depth that is deeper in the head. 

The continuous measurements taken during the febrile study, Figure 6, indicate that a maintainable, real-time accuracy of better than 0.5 °C is feasible over a lengthy period. The benefits of a continuously worn, accurate temperature measurement device could prove useful in numerous health and wellness scenarios such as fever and heat stroke detection, menstrual cycle tracking, and remote monitoring. Remote monitoring has the added benefit of providing safer, more cost-effective medical care, especially during pandemic or hospital bed shortage events. The device also has applications in monitoring the body temperature of individuals participating in high-physical exertion activities, such as competitive sports, or when they are working in high-temperature environments. 

Additionally, the device could be used in a variety of research studies where it is desirable to perform long-term, continuous body temperature monitoring using an unobtrusive, non-invasive device that can log and export data. The sensor can be worn comfortably all day with little occlusion affect due to its transparency mode and large venting system. The hearing aid also has an inertial measurement unit (IMU) that can be used to correlate activity level and type with body temperature. Finally, the battery allows the device to be used continuously for a day or longer depending on the hearing aid functions that are used.

Designing and building a continuously worn, accurate thermometer in a hearing aid platform already used by many people may be an ideal way to discreetly and conveniently perform routine vital sign monitoring. This technology could prove especially beneficial in the more elderly demographic of hearing aid wearers.

## 5. Conclusions

A dual temperature sensor system has been integrated into an all-day, ear-worn hearing device. Measurements along the temperature gradient of the ear canal were used to empirically fit a heat balance equation that was then used to estimate core body temperature. The predicted temperature values resulted in an intersubject LOA of ±0.49 °C over a variety of physiologic and ambient temperatures without calibration. The demonstrated accuracy and convenience of a continuously worn temperature measurement device could prove beneficial in several health and wellness situations such as fever detection and remote monitoring, especially in the elderly.

## Figures and Tables

**Table 1:** Bland–Altman Temperature Estimation Accuracy for Various Subject Groups.

Measurement Type	LOA (°C)	Measurements
**Indoor**	±0.51	127
**Outdoor**	±0.29	6
**Febrile**	±0.45	29
**Combined**	**±0.49**	**162**

## Data Availability

Not applicable.
